# What Does CATS Have to Do With Cancer? The Cognitive Activation Theory of Stress (CATS) Forms the SURGE Model of Chronic Post-surgical Pain in Women With Breast Cancer

**DOI:** 10.3389/fpsyg.2021.630422

**Published:** 2021-03-23

**Authors:** Alice Munk, Silje Endresen Reme, Henrik Børsting Jacobsen

**Affiliations:** ^1^The Mind-Body Lab, Department of Psychology, Faculty of Social Sciences, University of Oslo, Oslo, Norway; ^2^Department of Pain Management and Research, Oslo University Hospital, Oslo, Norway

**Keywords:** breast cancer, chronic postsurgical pain, cognitive activation theory of stress, expectancies, sickness behavior, stress, predictive coding, hypnosis

## Abstract

Chronic post-surgical pain (CPSP) represents a highly prevalent and significant clinical problem. Both major and minor surgeries entail risks of developing CPSP, and cancer-related surgery is no exception. As an example, more than 40% of women undergoing breast cancer surgery struggle with CPSP years after surgery. While we do not fully understand the pathophysiology of CPSP, we know it is multifaceted with biological, social, and psychological factors contributing. The aim of this review is to advocate for the role of response outcome expectancies in the development of CPSP following breast cancer surgery. We propose the Cognitive Activation Theory of Stress (CATS) as an applicable theoretical framework detailing the potential role of cortisol regulation, inflammation, and inflammatory-induced sickness behavior in CPSP. Drawing on learning theory and activation theory, CATS offers psychobiological explanations for the relationship between stress and health, where acquired expectancies are crucial in determining the stress response and health outcomes. Based on existing knowledge about risk factors for CPSP, and in line with the CATS position, we propose the SURGEry outcome expectancy (SURGE) model of CPSP. According to SURGE, expectancies impact stress physiology, inflammation, and fear-based learning influencing the development and persistence of CPSP. SURGE further proposes that generalized response outcome expectancies drive adaptive or maladaptive stress responses in the time around surgery, where coping dampens the stress response, while helplessness and hopelessness sustains it. A sustained stress response may contribute to central sensitization, alterations in functional brain networks and excessive fear-based learning. This sets the stage for a prolonged state of inflammatory-induced sickness behavior – potentially driving and maintaining CPSP. Finally, as psychological factors are modifiable, robust and potent predictors of CPSP, we suggest hypnosis as an effective intervention strategy targeting response outcome expectancies. We here argue that presurgical clinical hypnosis has the potential of preventing CPSP in women with breast cancer.

## Introduction

Chronic post-surgical pain (CPSP) affects a substantial amount of patients undergoing either major or minor surgeries (Shug and Pogatzki-Zahn, 2011). CPSP can be defined as pain that develops after surgical intervention and persists minimum 3–6 months after healed tissue damage (Cohen and Raja, 2020).

An example of debilitating CPSP is documented in women undergoing breast cancer surgery. More than 1 million women are diagnosed with breast cancer every year, and approximately 25–60% of them will struggle with CPSP, regardless of surgical procedure (Andersen and Kehlet, 2011; Wang et al., 2018b). The prevalence of severe CPSP following breast cancer surgery is estimated to be 5–10%, where CPSP causes patients to experience a significant reduction in daily functioning, work capability, and quality of life (Andersen and Kehlet, 2011).

As with any chronic pain condition, the pathophysiology of CPSP in breast cancer is multifactorial, and knowledge of the underlying mechanisms is still unclear. As an example of this complexity, only some of the women with CPSP following breast cancer surgery have peripheral pain drivers as a result of intra-surgical nerve damage (Gärtner et al., 2009; Schou Bredal et al., 2014). It is therefore acknowledged that CPSP is best understood through a bio-psycho-social model, with multivariate factors contributing to its development (Weinrib et al., 2017).

Some of the more established risk factors of CPSP includes pre-surgical stress-level, depression, anxiety, pain catastrophizing, and low optimism (Jackson et al., 2016; Weinrib et al., 2017; Jensen and Johannesen, 2019; la Cour, 2019; Giusti et al., 2021). Also, pre-surgical- or intense acute post-surgical pain can significantly increase the risk of CPSP in women with breast cancer (Gärtner et al., 2009; Schou Bredal et al., 2014).

When evaluating modifiable and well-documented risk factors for CPSP following breast cancer surgery, we argue for the potential impact of expectancies on psychoneuroimmunological responses to a stressful situation. Conceptualized by an expectancy model (SURGE), we propose that CPSP can be understood, delineated, and possibly prevented. Our suggested model incorporates the cognitive activation theory of stress (CATS), predictive coding principles, cortisol function, and inflammatory-induced sickness behavior.

## Setting the Scene for CPSP: Life Leading Up to a Surgery

Throughout our lives, our learning history shapes expectancies, higher order beliefs about how we will respond to stressful challenges such as an impending surgery. Surgery in the context of cancer represents a highly stressful experience for most, if not all. It gives rise to multitude of expectancies of how the surgery and disease will unfold and how one is going to deal with the consequences. Dealing with such a challenge evokes past learning in the form of acquired expectancies and prior conditioning, here seen as complementary and overlapping constructs (Stewart-Williams and Podd, 2004).

Expectancies are commonly defined as “beliefs that something will happen or is likely to happen” (Schwarz et al., 2016) and can be acquired by direct experience, verbal instruction, or observation of others (Kube et al., 2017; Laferton et al., 2017; Rief and Joormann, 2019). In other words, any direct or indirect experience with surgery will contribute to the formation of expectancies. The subsequent expectancies can be colored by hope, trust, and optimism, but also by fear, worry, and catastrophic thoughts. As an example, if a loved one previously has undergone surgery and experienced CPSP, we might fear an approaching surgery.

This fear quickly becomes important as expectancies can be the powerful modulators of health outcomes (Benedetti, 2008; Kirsch, 2018; Lasselin et al., 2018). Some of the strongest effects from expectancies are seen in the placebo/nocebo literature. Positive expectancies about a given treatment can lead to increased pain relief, even if the given treatment is perceived as inactive, e.g., a calcium tablet or sham acupuncture (Benedetti, 2008; Atlas and Wager, 2012; Forsberg et al., 2017). Also, it is well-established that positive expectancies about the response of a given treatment may enhance the analgesic effects of active surgical (Gandhi et al., 2009), pharmacological (Bingel et al., 2011), and non-pharmacological treatments (Peerdeman et al., 2016). These processes are coined placebo analgesia. A related phenomenon is nocebo hyperalgesia. Here, negative response outcome expectancies are found to increase the intensity of pain in experimental and clinical studies (Colloca and Miller, 2011; Petersen et al., 2014). Negative expectancies about a treatment can block the analgesic effects of active treatments or exaggerate negative side effects (Petersen et al., 2014; Smith et al., 2020). While most of this research primarily focuses on experimental and acute pain, other lines of research have shown how negative expectancies can have debilitating effects on the development and maintenance of chronic pain (Atlas and Wager, 2012).

## Cognitive Activation Theory of Stress

From the moment an individual receives word about an upcoming surgery, particularly, a potential life-threatening cancer requiring surgery, a stress response usually follows. This response can be understood using the cognitive activation theory of stress (CATS), a psychobiological theoretical framework offering clear and formal definitions of the stress response and how this affects health (Ursin and Eriksen, 2004).

**Figure 1:**
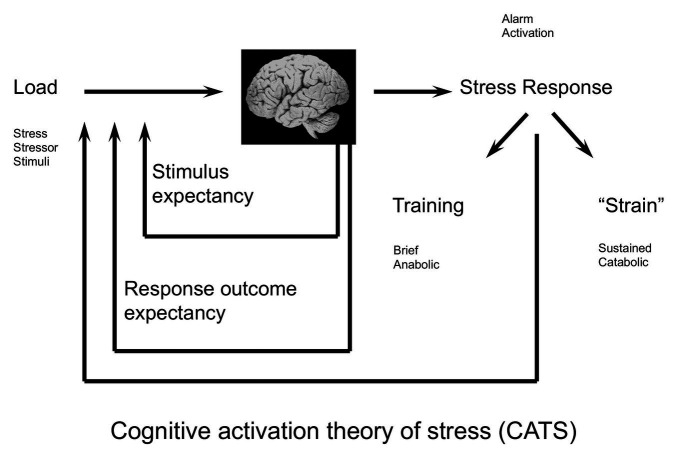
The cognitive activation theory of stress (CATS; Ursin and Eriksen, 2010). The stress stimulus (load) is registered. Stimulus- and response- outcome expectancies influence whether the load is appraised as stressful. If so, a general physiological stress response is activated. Feedback from the physiological stress response is being fed back to the brain. A short activation of the stress response is healthy and adaptive, while a sustained stress response may lead to illness or disease. Reprinted from Ursin and Eriksen (2010), Copyright (2021) with permission from Elsevier.

In CATS, “stress” is defined and operationalized as a psychobiological concept with four stages (Figure 1). The first stage is the orientation. Here, we orient toward what could be a *stress stimulus*, representing objective internal or external stimuli automatically processed by the brain, ultimately leading to appraisal.

The second stage is the appraisal or subjective anticipation of stress, where the stimuli have been filtered by the brain in terms of individual learning history. In CATS, learning history includes stimuli expectancies driven by classical conditioning, and response outcome expectancies driven by operant conditioning. These expectancies determine to a large degree intensity and duration of the third stage, the physiological stress response.

The physiological stress response is an alarm system representing a general, non-specific arousal response in the somatic and autonomic nervous system as well as in several endocrine axes (Ursin and Eriksen, 2010). The alarm goes off when an imbalance is expected in the homeostatic system, e.g., when experiencing novel or threatening stimuli or a discrepancy between what is expected and what actually is (Subjective set Value − Actual Value ≠ 0; Ursin and Eriksen, 2010).

The fourth and final stage of the definition represents the individual experience of the stress response, consisting of information from the arousal response being fed back to the brain, ultimately maintaining, adding to, or resolving the unpleasant feeling of stress.

According to CATS, stress is a beneficial reaction, meaning that an activation of a stress response in challenging situations is healthy and adaptive. The goal of a short activation of the physiological stress response is to restore homeostasis (Ursin and Eriksen, 2004), and the arousal response is gradually turned off when the individual expects to handle the challenge successfully. If not, the arousal may be sustained, leading to illness and disease. Whether the stress response is eliminated, dampened, or sustained relies on expectancy filters (Ursin and Eriksen, 2004). These filters are described as stimulus expectancies and response outcome expectancies.

### Stimulus Expectancies

Our brain is designed to store information about the relationships between sets of stimuli and our available responses (Ursin and Eriksen, 2004). This information is stored as expectancies and is how we come to expect that one specific stimulus typically precedes another specific stimulus. In CATS, this is called as stimulus expectancies, and it represents classical conditioning within traditional learning theory (Ursin and Eriksen, 2004). A classic example of associative learning and stimulus expectancies is the work of Ivan Pavlov and his dogs. His now famous experiment showed how dogs that were continuously presented with food paired with a sound of the bell later would salivate when they heard the bell ring, regardless of food were offered or not (Pavlov and Thompson, 1902). A particular feature of Pavlovian conditioning is that stimuli sharing characteristics with the original conditioning stimuli may become capable of eliciting conditioned responses, depending on the perceptual or functional proximity between the two. This was exemplified in his studies showing that the dogs eventually started to salivate just as they heard the footsteps of the experimenters. Thus, during stimulus generalization, individuals extrapolate knowledge from one aspect of the situation to other aspects and situations – making more and more stimuli capable to elicit the conditioned response.

### Response Outcome Expectancies

Response outcome expectancies are within CATS regarded as acquired information about available responses to a stimulus and how these responses affect subsequent outcomes. This type of learning follows principles of operant or instrumental conditioning, where the individual learns from positive and negative reinforcements of behavior (Ursin and Eriksen, 2004, 2010). Through response outcome expectancies, you anticipate successful or unsuccessful handling of future threats without yet having experienced them, an essential prerequisite for avoiding or anticipating harm.

A physiological stress response experienced by a woman who is about to undergo surgery could thus be interpreted in different ways according to her expectancies; it could either be interpreted as a sign of anxiety implicating uncontrollable danger and harm, or as a normal response to a challenging situation. While the first interpretation has the potential to increase and sustain the stress activation, the second interpretation has the potential to dampen the stress response.

The power of beliefs and expectancy in regulating physiology is a hallmark of another important learning theory, the predictive coding framework of information processing. This theory suggests that the brain uses Bayesian prediction principles to constantly match bottom-up sensory information with top-down predictions created by prior experiences (Gilbert and Sigman, 2007; Petrovic et al., 2010; Büchel et al., 2014). These predictions are organized hierarchically in the brain, from lower-level momentary hypotheses about the causes of current sensory inputs (e.g., feeling pain from a gentle touch) to increasingly more overarching beliefs the nature of the world and yourself (e.g., “I cannot cope with this pain anymore”). These higher order beliefs are in many ways analog to the concepts of stimulus expectancies and response outcome expectancy in CATS.

One can envision generalized response outcome expectancies forming enduring overarching hypotheses (e.g., “I am not a person that handles pain”). When a person experiences a discrepancy between experience and expectancy, these higher order beliefs can overturn lower-level sensory input, motivating behavior and cognition in order to uphold an expectancy, regardless of lower level input and prediction errors. This has been described as cognitive immunization and is particularly evident in patients with depression (Kube et al., 2020). Numerous studies show how patients suffering from depression are prone to maintain their negative expectancies despite of positive, contradictory evidence (Korn et al., 2014; Liknaitzky et al., 2017; Everaert et al., 2018). This immunization contrasts that of a healthy population who show an overall optimism bias, i.e., a tendency mainly to update expectancies if new information are positive, while maintaining one’s prior belief if the presented evidence is negative (Sharot, 2011).

The notion of a hierarchical organization of processing is also described in CATS through the feedback loop in the model, where lower level peripheral changes – i.e., the stress response – is being fed back to the brain, but can be prolonged or dampened according to higher order expectancies or predictions (Ursin and Eriksen, 2004). Principles from the predictive coding framework thus align with the expectancy principles outlined in CATS. In effect, generalized expectancies based on prior experiences can then override lower-level changes and new learning, potentially maintaining a stress response in the weeks leading up to breast cancer surgery. The CATS model has further specified three forms of generalized response outcome expectancies, namely coping, helplessness and hopelessness.

#### Coping

A significant contribution from CATS is its clarification of the coping term and its assumed correlates. Coping in CATS terminology is the acquired expectancy that most or all responses to a situation will lead to a positive outcome. Thus, it represents an anticipatory cognitive construct rather than objective abilities or strategies that could be applied in challenging situations. Coping in form of generalized response outcome expectancy may be associated with a proactive appraisal of the stressful situation, reflecting improved anticipatory stress regulation, ultimately resulting in a shortened physiological stress response (Ursin and Eriksen, 2004).

In the case of a woman undergoing breast cancer surgery, coping may refer to the expectancy of being able to handle the stressful aspects of the surgery, i.e., the post-surgical pain and potential side effects in a successful way. This taps into the established CPSP resilience factors of dispositional optimism (Powell et al., 2012) and self-efficacy (Weinrib et al., 2017).

According to CATS, it is when coping is defined as a generalized response outcome expectancy it may hold the strongest predictive power for health outcomes, mediated by its presumed reducing effects on the strength and the duration of the physiological stress response (Ursin and Eriksen, 2004, 2010). The authors of CATS argue that since *coping defined as coping strategies* can be carried out under various levels and lengths of arousal, it is not a robust predictor of stress-related illness or disease (Ursin and Eriksen, 2004).

Both human and animal studies suggest that positive expectancy attenuates the cortisol response to stress. Rats exposed to shocks will initially show high behavioral and endocrine arousal. However, in late stages of avoidance learning tasks when they have established that they will be able to escape the shocks, the arousal diminishes to a minimum (Coover et al., 1973). Ursin and Eriksen (2004) suggest that this happens so rapidly and efficient that it is not just a result of the avoidance behavior, but due to an expectancy that the behavior will lead to a successful outcome.

Ursin et al. (1978) also tested this position in humans. A group of novel parachutist trainees showed the high levels of endocrine and subjective reported arousal before their first jump. Already after their first training session, before there had been any real improvement of their performance, the arousal reduced significantly. This could indicate that it was not the actual performance, but the acquired expectancy of being able to handle the situation with a positive result, that explained the diminished stress response.

Recent studies of how we react to psychosocial stress confirm and expand upon these early reports of positive physiological effects from cognitive re-framing and coping. Jamieson et al. (2012) showed that during a psychosocial stress test, participants instructed to reappraise their arousal in a positive way had increased cardiac efficiency, lower vascular resistance, and decreased attentional bias. Similarly, Nasso et al. (2019) showed that when anticipating a stressful task (i.e., giving a speech), individuals using an adaptive cognitive emotion regulation strategy showed better anticipatory stress regulation than individuals prone to worry or catastrophizing. Overall, these results suggest that positive response outcome expectancies can affect the long-term consequences of our physiological stress responses in a beneficial fashion.

#### Helplessness and Hopelessness

Helplessness refers to the acquired expectancy of one’s actions having no impact on the outcome of an aversive event. This can be exemplified by a woman going into breast cancer surgery with the expectancy that there is nothing she can do to control the outcome of the surgery or potential negative side effects. A qualitative study by Lie et al. (2018) highlighted how young adult cancer patients, aged 18–35 years at time of diagnosis, describe that not being able to predict or control their situation was the most stressful aspect of all stages of their disease and treatment. This study focuses on patients in a particular vulnerable transitional life period. However, other studies find similar results on helplessness, i.e., the factors of uncertainty and lack of perceived control are common characteristics of stress and chronic disease, with negative effects on pain outcomes and quality of life (Johnson et al., 2006; Müller, 2011; Caruso et al., 2014; Engevold and Heggdal, 2016).

Hopelessness, on the other hand, is an expectancy of most or all responses leading to negative outcomes. In women with breast cancer going into surgery, this could be the expectancy that all attempts to handle or change the stressful situation evolving around the surgery, will only make it worse. Hopelessness implies that there is control, responses have effects, but they are all negative. These failed attempts combined with the assumed control could evoke feelings of guilt and self-blame in those who acquire expectancies of hopelessness. Thus, these expectancies are proposed by the authors of CATS as a cognitive model for depression (Ursin and Eriksen, 2004), a condition that increases the risk of developing CPSP (Weinrib et al., 2017).

The expectancies of helplessness and hopelessness are also conceptually close to another established risk factor of CPSP namely pain catastrophizing. When measured with the Pain Catastrophizing Scale (PCS; Sullivan et al., 1996), this is a strong and consistent predictor of CPSP (Hannibal and Bishop, 2014; Johannsen et al., 2018, 2020). In PCS, patients report about helplessness and hopelessness in response to pain (e.g., “It’s terrible and I think it’s never going to get any better” and “there’s nothing I can do to reduce the intensity of the pain”; Sullivan et al., 1996). Moreover, the elements of hopelessness are captured within measures of injustice experiences (The Injustice Experience Questionnaire; Sullivan et al., 2008), which also is a significant psychological risk factor for developing CPSP (Yakobov et al., 2014).

In summary, CATS states that coping may reduce or eliminate the physiological stress response, and helplessness and hopelessness may sustain it. If sustained, the stress response affects specific psychological and neurobiological mechanisms that can reinforce and perpetuate pain relating to the surgery, increasing the risk for developing CPSP.

### Stress and Sensitization

A line of experimental studies have demonstrated the link between a sustained stress response and the process of sensitization, which is suggested as a psychobiological mechanism in the transition from acute to chronic pain (Ursin, 2014). On the cellular level, sensitization is defined as an increased efficiency in a neural circuit, due to a change in synapses from repeated use (Collingridge et al., 2004). Sensitization of pain pathways in the central nervous system is widely accepted as a theory of neural mechanisms enhancing pain transmission (Ikeda et al., 2009). This central sensitization progressively amplifies the responses to pain stimuli. It manifests as pain hypersensitivity both as a reduction in pain threshold and an increase in pain responsiveness as well prolonged after sensations and an expansion of the receptive field (Woolf, 2011).

A large body of evidence showing central sensitization in chronic pain syndromes originates from research on patients with fibromyalgia, a condition with widespread pain in the body. Research has demonstrated widespread reductions in pain thresholds as well as an increased temporal summation and a spatial area of pain in this patient group (Gibson et al., 1994; Lorenz et al., 1996; Graven-Nielsen et al., 2000). Patients with CPSP also show the signs of central sensitization (Woolf, 2011; Johannsen et al., 2020). The role of central sensitization in CPSP is further supported by an indication of pain reducing effects due to centrally acting agents such as ketamine (Remérand et al., 2009), pregabalin (Mathiesen et al., 2009; Burke and Shorten, 2010), gabapentin (Sen et al., 2009; Verret et al., 2020), and duloxetine (Ho et al., 2010). However, more studies are needed to establish the effectiveness of pharmacological treatments.

Pre-surgical pain in the surgical area as well as other sites of the body is the strong predictors of CPSP (Poleshuck et al., 2006; Kudel et al., 2007; Gärtner et al., 2009; Nikolajsen and Aasvang, 2019). Patients who experience pre-surgical pain conditions, such as fibromyalgia, migraine, or chronic low back pain, have a significant increased risk of CPSP following breast cancer surgery (Bruce et al., 2012; Schou Bredal et al., 2014). The association between pre- and post-surgical pain could be due to an unknown common underlying factor (e.g., genetic and/or psychological), making a group of patients more vulnerable to persistent pain. Still, it could suggest that a central sensitization plays a role in CPSP through repeated pain stimuli increasing the efficiency and excitability of central pain pathways or stated another way; pain produces pain.

Different lines of research thus present the hypothesis that sensitized stress responses could interact with sensitized pain responses, and ultimately increase the risk of CPSP. However, it has proven difficult to establish direct causal delineation of sustained stress in chronic pain, but psychological and physiological stress is associated frequently with the development and persistence of chronic disease such as chronic pain conditions (McEwen and Kalia, 2010; Timmers et al., 2019). In a CATS perspective, sustained activation is the motor that accelerates sensitization and prevents its reversibility, thus sustained stress activation will affect almost all bodily systems through the actions of cortisol. As principles of central sensitization likely contribute to the chronification of pain (Woolf, 2011), the potential maladaptive effects of stress hormones on pain transmission could mediate the relationship between chronic stress and chronic pain.

### Cortisol Function and Chronic Pain

Cortisol is a catabolic hormone produced in the adrenal cortex, which plays a crucial part in the physiological stress response (Hannibal and Bishop, 2014). In stressful situations, cortisol levels rise to provide energy to deal with the situation or escape danger (fight or flight; Blackburn-Munro and Blackburn-Munro, 2003). Prolonged cortisol secretion, on the other hand, could have damaging effects and increase the risk of chronic pain.

During the stress response, unbound cortisol binds on glucocorticoid receptors (GRs) resulting in anti-inflammatory and pain inhibiting mechanisms (Fries et al., 2005; Sorrells et al., 2009). However, an exaggerated or sustained cortisol secretion may cause GR to downregulate, or block cortisol binding, ultimately creating cortisol dysfunction (Norman and Hearing, 2002). Further, an impaired binding to GR might disrupt the negative feedback loop, which under normal circumstances enables cortisol to regulate the release of corticotrophin-releasing hormone (CRH) (Fries et al., 2005). CRH upregulates glutamate and N-methyl-d-aspartate (NMDA) in the amygdala, which might set prime for a conditioned fear-based stress response (Tsigos and Chrousos, 2002; Shekhar et al., 2005; McEwen and Kalia, 2010). Additionally, it is indicated that the activation of CRH receptors in the amygdala may trigger pain in the absence of tissue damage and that hyperpolarized postsynaptic potentials might be able to make amygdala resistant to inhibitory signals from prefrontal cortex (Shekhar et al., 2005; Ji et al., 2013). Such reduced prefrontal modulation is associated with pain catastrophizing in chronic pain patients experiencing intense pain (Seminowicz and Davis, 2006).

Several studies have associated the actions of cortisol with increased activation in the amygdala during anxiety and fear (Shekhar et al., 2005; Ji et al., 2013; Vachon-Presseau et al., 2013). Using an animal model of neuropathic pain in rats, Li et al. (2013) found that lesions of the basolateral amygdala inhibit the transition from acute to chronic pain in the early stages of nerve damage. Due to the well-established role of the amygdala in the fear learning system, the authors suggest that a possible explanation of this involves interruptions of negative emotions and consolidation of fear-based pain memories. Such learning processes may possibly relate to the acquisition of negative response outcome expectancies, potentially leading to sustained activation, sensitization and chronic pain.

Pain catastrophizing, i.e., a sense of helplessness and hopelessness, elevates the cortisol secretion and sustains the activation of the stress response (Johansson et al., 2008; Quartana et al., 2010; Müller, 2011). Sustained activation of a sensitized stress response exhausts the HPA-axis, and chronic stress-induced hypocortisolism has been linked to chronic pain conditions (Tsigos and Chrousos, 2002; Tak and Rosmalen, 2010; Hannibal and Bishop, 2014). Paradoxically, hypercortisolism is also reported as a contributor to chronic pain (Blackburn-Munro and Blackburn-Munro, 2003; Dedovic et al., 2009), i.e., potentially mediated by the blunted feedback mechanisms discussed earlier in this section. The relationship between stress, chronic pain, and hypo- and hyper-cortisolism thus depend on temporal aspects of measurement, the individualized stress response, the different mechanisms of cortisol dysfunction described earlier and numerous situation-specific factors (Hannibal and Bishop, 2014). These inconsistencies call for more research on the relationship between cortisol and chronic pain, but available data suggest that stress-induced cortisol dysfunction could contribute to the development and persistence of chronic pain.

Cortisol dysfunction through the mechanisms discussed above represents potential harmful effects of sustained activation on a neurochemical level. In addition, prolonged secretion of stress hormones may alter both the functional and physical properties of the corticolimbic system with considerable consequences for the development and perpetuation of chronic pain following breast cancer surgery.

### Corticolimbic Plasticity

The corticolimbic circuit of the brain consists of neural loops between structures such as the prefrontal cortex (PFC), the amygdala, the hippocampus, and hypothalamus in strong connections to the HPA-axis (Vachon-Presseau, 2018). The corticolimbic circuit is involved in a variation of cognitive-emotional processes and plays a crucial role in motivation and learning, i.e., in relation to pain and the anticipation of pain (Ploghaus et al., 2001; Apkarian et al., 2009). It has been suggested that the corticolimbic circuit may represent the primary system through which nociception accesses consciousness and is experienced as pain (Baliki and Apkarian, 2015). The corticolimbic structures show high affinity to stress hormones, which enable them to regulate the stress response through feedback loops to the HPA axis, and at the same time making them sensitive to the effects of long-term exposure to cortisol (Radley and Sawchenko, 2011; Vachon-Presseau, 2018).

The PFC is particular sensitive to the effects of stress hormones. Sustained exposure to cortisol has shown to generate extensive dendritic spine loss (Arnsten, 2009; McEwen and Morrison, 2013) similar to that observed in medial prefrontal cortex (mPFC) in animal models of neuropathic pain (Metz et al., 2009). Moreover, the mPFC has been associated with individual differences in subjective pain intensity in chronic pain patients. For example, an fMRI study by Baliki et al. (2012) indicated that the strength of the functional connectivity between mPFC and nucleus accumbens (NAc) is a dominating predictor of pain chronification in humans with subacute back pain (stronger mPFC-NAc connectivity was associated with pain persistence). The activity of the PFC regulates, and is regulated by, the amygdala. In animal models of chronic pain, the excitability of neurons in the amygdala rapidly increases in response to repeated pain stimuli (Ursin, 2014). This increased excitability compliments animal models showing hypertrophy and increased spinogenesis in basolateral regions of the amygdala when animals are exposed to sustained stress (Roozendaal et al., 2009). Studies of post-traumatic stress disorder in humans expand upon this indicating that both pain and fear-based learning can drive hypertrophy in these regions of the amygdala (Morey et al., 2020). The increased activity and hypertrophy of the amygdala divergently affects plasticity in other brain regions such as the PFC and hippocampus (Patel et al., 2018). The amygdala then influences the corticolimbic circuit by modulating excitability of the inhibitory neurons in the mPFC, as well as neurons in the spinal cord (Neugebauer et al., 2004; Neugebauer, 2015), which may result in pain hypersensitivity. Thus, the connectivity between the amygdala and the PFC may be distorted by long-term exposure to cortisol, mediated by CRH as well as GR signaling (Galatzer-Levy et al., 2017), which have implications for the regulation of anxiety and pain (Shekhar et al., 2005; Ji et al., 2013).

Finally, several studies have implicated that alterations in the physical and functional features of the hippocampus are associated with chronic pain conditions. Using an animal model of neuropathic pain, Mutso et al. (2012) found decreased hippocampal neurogenesis and altered hippocampal short-term synaptic plasticity in mice with spared nerve-injury neuropathic pain compared with sham animals. In addition, this study found lower hippocampal volume in patients suffering from low back pain and complex regional pain syndrome. The authors propose that the functional hippocampal abnormalities found in their animal model of neuropathic pain potentially relate to the decreased hippocampal volume observed in chronic pain conditions, and that this ultimately contributes to emotional and learning deficits associated with chronic pain. The deteriorating effects of stress hormones on hippocampal volume and neurogenesis are indicated in both aging (Lupien et al., 1998) and psychiatric populations (Sapolsky, 2000; Videbech and Ravnkilde, 2004).

In summary, the corticolimbic system may be sensitive to maladaptive effects of long-term exposure to stress hormones, both in terms of its physical and functional properties. These stress-induced changes in the corticolimbic circuit may negatively affect the regulation of the stress response by impairing the inhibitory feedback loops from the HPA axis (Vachon-Presseau, 2018). This could contribute to a vicious cycle sustaining the activation of the stress response and presents direct and indirect implications for the chronification and experience of pain in a woman entering surgery for breast cancer.

## Peri- and Postoperative Stress – the Crucial Time Just Before, During, and After Surgery

In the perioperative phase, breast cancer patients often experience high levels of distress and expect a variety of post-surgery symptoms (Deane and Degner, 1998; Spencer et al., 1999). Such distress may include everything from concerns about diagnosis and prognosis (Schnur et al., 2008), to concerns about anesthesia (Shevde and Panagopoulos, 1991), and surgical procedures (e.g., pain during procedure and postoperative side effects; Klafta and Roizen, 1996). Pre-surgery distress and patient expectancies about the severity of postoperative side effects have both been found to predict pain severity, nausea, and fatigue 1 week after surgery in breast cancer patients (Montgomery et al., 2010). In addition, patients’ presurgical expectancies of pain, fatigue, and nausea have been shown to partially mediate the effects of distress on pain severity 1 week after surgery, where expectancies and psychological distress together explained 28% of the variance in 1 week post-surgery pain (Montgomery et al., 2010). In CATS terminology, this would entail background arousal (high distress), stimulus expectancies (severe pain from surgery), and response outcome expectancies (“I have no power over what’s to come”), resulting in a tonic (sustained) arousal with increased risk of negative health consequences (e.g., pain and other side effects 1 week after surgery).

A breast cancer surgery usually involves either total removal of the breast (mastectomy) or breast-conserving surgery (lumpectomy) with or without sentinel node biopsy. Breast conserving surgery is a less invasive yet a safe and effective option (Fisher et al., 1989) and is the most commonly performed surgery (Lazovich et al., 1999). While breast conserving surgery has fewer early post-operative complications (Chatterjee et al., 2015) and has been associated with better quality of life (Sun et al., 2014), incidence rates of CPSP appear to be less influenced by type of surgery (Wang et al., 2016). Instead, CPSP is heavily influenced by emotional distress, which has led to a general call for ways to target the emotional distress, since this is a modifiable risk factor that could be intervened on (Jackson et al., 2016). In a recent study, those women with the highest level of distress after surgery were those who benefited the most from a psychological treatment (Wang et al., 2018a,b). We therefore argue that from a prevention perspective, timing of the intervention is crucial. The time window immediately before surgery, on the day of surgery, is critical. If distress is reduced and coping increased already prior to surgery, an important risk factor for CPSP and other negative health outcomes could be eliminated, ultimately affecting the prognosis and risk for CPSP.

### Inflammation, Sickness Behavior, and Post-surgical Pain

Stress, inflammation, and pain are inherently interlinked systems, whether you look at it from an acute or chronic perspective. Both pro- and anti-inflammatory processes kick in, in response to stressors such as pain, perceived or anticipated danger, injury, and infection (Slavich and Cole, 2013). A short-term pro-inflammatory response increases the chance of survival by accelerating wound healing and limit potential spread of an infection. In addition, pro-inflammatory cytokine activity, involving tumor necrosis factor-*α* (TNF-α) and interleukins 1*β* and 6 (IL-1β and IL-6), promote a distinct motivational state called as sickness behavior, observed in both human and animals (Hart, 1988; Dantzer, 2001; Shattuck and Muehlenbein, 2016).

The cluster of behavioral symptoms that constitutes sickness behavior includes fatigue, pain hypersensitivity, psychomotor retardation, social withdrawal, and decreased interest in hedonic behaviors (Dantzer, 2001; Lasselin et al., 2020). Sickness behavior also involves an emotional component, i.e., heightened emotional distress, which are evident in humans exposed to experimentally induced inflammation (Lasselin et al., 2018). This motivational state lowers (social) activities in order to facilitate recovery and decrease the risk of spreading an infection to conspecifics. In addition, hypervigilance involving pain hypersensitivity and emotional distress would motivate the vulnerable organism to tend to one’s wounds and stay away from potential danger while recovering.

As with the stress response, a short increased inflammation and subsequent sickness behavior is adaptive and desirable. However, the inflammation and sickness behavior need to subside for health and healing to take place. Unfortunately, fear-based learning, threat monitoring (i.e., searching for pain in the area of surgery), and sustained stress can maintain inflammation processes through stress-driven alterations in the nucleuses of the amygdala. Recent imaging studies in humans have shown how a hyperactive amygdala activates leukopoietic tissue in the bone marrow, increasing arterial inflammation (Tawakol et al., 2019) and C-reactive protein (CRP) (Osborne et al., 2020). Elevated levels of CRP are strongly associated with reduced pain tolerance and increased pain sensitivity (Schistad et al., 2017), and increased pain sensitivity would increase acute post-operative pain, furthering the risk of developing CPSP (Wilder-Smith, 2011).

Increased inflammation in persistent pain also has a behavioral analog. Using a cross-sectional design, Jonsjö et al. (2020) concluded that chronic pain patients report high levels of sickness behavior (assessed with a validated questionnaire for subjective sickness behavior, Sickness Q; Andreasson et al., 2018). The level of sickness behavior in chronic pain patients was similar to the levels reported by healthy volunteers following injection with a lipopolysaccharide (LPS), a method used to induce a strong inflammatory response in human or animals (Jonsjö et al., 2020; Lasselin et al., 2020). LPS-injected individuals report higher pain sensitivity compared to controls, and the increase in pain sensitivity correlates with lower activation in the ventrolateral prefrontal cortex and the rostral anterior cingulate cortex – areas associated with top-down pain modulation (Karshikoff et al., 2016). Moreover, when compared to others, the levels of self-reported sickness behavior in chronic pain patients and LPS-injected individuals are significantly higher than general care patients and healthy subjects (Jonsjö et al., 2020).

In sum, when undergoing breast cancer surgery, the surgery naturally and adaptively elicits stress-, immune-, and pain-responses. Inflammatory-induced sickness behavior serves adaptive and protective functions in the acute post-surgical phase. However, if the women undergoing surgery enter and exit the surgery with brain alterations and increased inflammation driven by a sustained stress response, this could result in pain hypersensitivity and hypervigilance toward pain following in the weeks after surgery. This fits with persistent sickness behavior mirroring these alterations. While neural and humoral pathways that restore homeostasis may terminate sickness behavior, the same sickness behavior processes can be maintained without an ongoing infection (Jonsjö et al., 2020), possibly through inflammation driven by a sustained stress response.

Significantly elevated levels of the pro-inflammatory cytokine interleukin 6 (IL-6) are found in chronic pain patients compared to healthy controls (Koch et al., 2007). In addition, increased plasma concentrations of TNF-α and IL-1β, other common markers of low-grade systemic inflammation, were detected in chronic pain patients with severe pain, though not in patients with light or moderate pain, suggesting a potential role of low-grade inflammation in chronic pain at least when pain intensity exceeds a certain threshold (Koch et al., 2007). Overall, higher plasma concentrations of inflammatory markers correlate with higher self-reported pain intensity (Koch et al., 2007). As cytokines are thought to be the main mediators in this stress-induced pro-inflammatory effect, this has led to low grade pro-inflammatory processes being proposed as a biological mechanism directly contributing to the pathophysiology of stress-related diseases (Rohleder, 2014).

The previous sections have discussed various mechanisms through which sustained stress activation may contribute to CPSP following breast cancer surgery. Sickness behavior, cortisol dysfunction, and alterations in the corticolimbic circuit due to prolonged secretion of cortisol are essential. They combine to drive the physical and functional irregularities characteristic for chronic pain states, as evident in human and animal studies. Moreover, disrupted corticolimbic connectivity has negative consequences for the regulation of the HPA-axis through its inhibitory feedback loops. The potential maladaptive effects of long-term exposure to stress hormones are important aspects of the vicious cycle of chronic stress and chronic pain, preventing the “alarm” to be turned off, and enabling the stress and pain to persist many years after surgery.

Common to the proposed pathophysiological mechanisms of a stress-induced transition from acute to chronic pain is the involvement of various forms of learning. The corticolimbic circuit, in particular, the amygdala and the hippocampus, is essential in learning and consolidation of fear-based memories, i.e., in response to pain (Hannibal and Bishop, 2014; Vachon-Presseau, 2018). This contributes to the conditioning of a sensitized stress response, readily activated in response to pain (Hannibal and Bishop, 2014). It seems reasonable to hypothesize, that the corticolimbic pathways play a role in the conditioning of response outcome expectancies. Helplessness and hopelessness in response to pain relate to outcomes with strong affective value during high arousal, which make it likely to involve activation of limbic pathways. Furthermore, the relationship is likely bidirectional, in such that hopelessness and helplessness sustain the stress response and contribute to the long-term exposure and maladaptive effects of stress hormones on the corticolimbic circuit.

## The Surge Model

According to SURGE, generalized response outcome expectancies in form of helplessness and hopelessness sustain a physiological stress response before and after surgery. This sets the stage for fear-based learning, pain sensitization, and maladaptive effects from stress hormones. Moreover, the sustained stress response may contribute to increased pro-inflammatory activity in the peri- and post-operative phase. An increased and prolonged inflammatory state may lead to chronic sickness behavior with its characteristic cluster of hyperalgesia, emotional distress, and other debilitating behaviors.

**Figure 2:**
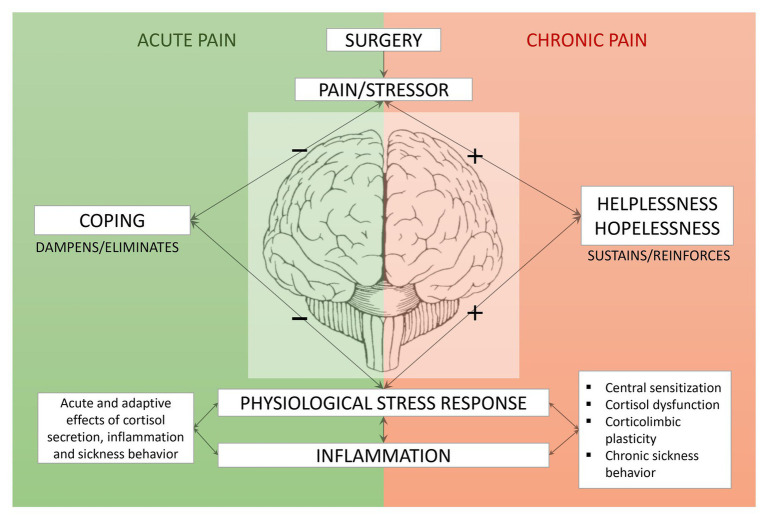
The SURGE Model of chronic post-surgical pain in women with breast cancer: surgery activates the central nervous system and creates acute pain. In line with CATS- and predictive-coding framework principles, the pain is appraised based on previous experiences in form of response outcome expectancies. An expectancy of being able to handle the pain with a positive outcome (coping) dampens or eliminates the physiological stress response. An expectancy of not being able to control or influence the pain (helplessness) or only making the pain worse (hopelessness) sustain the activation of the stress response. The sustained activation creates a vicious cycle of chronic stress, chronic inflammation, and chronic pain mediated by pathophysiological mechanisms such as central sensitization, cortisol dysfunction, impairment of corticolimbic connectivity, and inflammatory-induced sickness behavior.

We here propose that the SURGE model of CPSP (Figure 2) offers a possible explanation on how acute pain following breast cancer surgery may develop into CPSP depending on generalized response outcome expectancies. The model further proposes which psychobiological mechanisms drive this transition in form of a sustained activation of the stress response and inflammatory processes. Moreover, the model suggests targets for interventions that could prevent the development of CPSP in women with breast cancer.

## How to Address the Problem: Managing Expectancies

If response outcome expectancies are an important driver in the development of CPSP, mediated by sustained stress activation, a change in these expectancies should be followed by reduced stress activation and a correspondingly reduced risk of acute as well as CPSP. Challenging and changing negative expectations are fundamental to several psychological interventions. In cognitive behavioral therapy (CBT), unhelpful cognitions are targeted and challenged with a goal of reversing thoughts of helplessness and hopelessness (Beck and Dozois, 2011). The efficacy of CBT has been demonstrated in several populations and settings, including women with breast cancer (Antoni, 2013), with evidence from self-reported outcomes as well as cancer-relevant biological outcomes (McGregor and Antoni, 2009). A more recent approach from the third generation CBT is Acceptance and Commitment Therapy, which also holds promise as a valuable adjunct to surgical interventions (Weinrib et al., 2017).

Nevertheless, in the myriad of psychological interventions and techniques, one particular intervention stands out as notably potent in the context of surgery namely clinical hypnosis. Verbal suggestions appear to be a particularly powerful way of changing expectancies, and this very element is refined and perfected in hypnosis. The seminal study by Montgomery et al. (2007) demonstrates the effects of a hypnosis in women undergoing breast cancer surgery, where a brief session of hypnosis focusing on increasing coping expectancies right before surgery, produced large reductions in pain, distress, and discomfort immediately after surgery.

Hypnosis has been defined in various ways, but is most often described as a state of highly focused attention and increased suggestibility (Lynn et al., 2010). It is often compared to the everyday state of becoming so immersed in a good book or a movie that you enter the imagined world and loose contact with the real world (Lang et al., 2000; Montgomery et al., 2002).

The evidence-base for clinical hypnosis as an effective adjunctive non-pharmacological analgesia is strong, as demonstrated in several articles and meta-analyses in top-tier journals (Lang et al., 2000; Montgomery et al., 2002; Tefikow et al., 2013; Kekecs et al., 2014). Of particular relevance here are effects that involve pain reduction, reduced need for medication, and shorter duration of surgery, with effect sizes indicating better clinical outcomes in patients receiving hypnosis than 89% of patients in control groups (Montgomery et al., 2002). Hypnosis has further been shown to be superior to other psychological techniques (e.g., therapeutic suggestions; Kekecs et al., 2014) and might also provide benefits when delivered *during* general anesthesia (Berlière et al., 2018; Lacroix et al., 2019; Nowak et al., 2020). While studies of long-term effects of hypnosis are scarce, one recent study indicates the potential for preventing CPSP with peri-operative hypnosis (Berlière et al., 2018) in line with the SURGE model of CPSP.

### Mechanisms of Hypnotic Analgesia

Exactly how hypnotic analgesia works is heavily debated and not agreed upon. While some insists that hypnosis involves an altered state of consciousness (Lynn et al., 2010) others refer to hypnosis as a cognitive behavioral technique (Montgomery et al., 2007), implying that it works through the same system as placebo analgesia works through. Our approach is mostly in line with the latter position. Consistent with the SURGE model, we propose that hypnotic analgesia might work through hypnotic suggestions inducing positive coping expectancies in response to surgery and pain, leading to a dampening of the physiological stress response and ultimately a decrease in pain intensity and a lower risk of developing CPSP.

Nevertheless, earlier studies have demonstrated that hypnotic analgesia could occur through other systems than through the endogenous pain inhibitory mechanisms within the central nervous system that placebo works through (Barber and Mayer, 1977). Injections of naloxone, which is an opioid antagonist, have for instance not been able to change the elevated pain threshold induced by hypnosis in acute (Barber and Mayer, 1977) or in chronic pain (Spiegel and Albert, 1983).

Rather than a placebo effect “in disguise,” or an altered state of consciousness, we argue that hypnotic analgesia instead involves an altered perception. This has been suggested by leading experts in the field (Spiegel, 2007) and aligns well with the SURGE model. Through a mobilization of attention pathways in the brain brought about by hypnosis, specific instructions are given that alters the experience of pain and associated anxiety.

The recent predictive coding approaches have also shown relevance to hypnosis. By suggesting that hypnosis causes a shift in the default mode network (DMN; Carhart-Harris and Friston, 2019), an opportunity is created for the psychotherapeutic context surrounding the administration to establish longer-term changes in predictive coding activity. By increasing their sensitivity toward prediction errors, otherwise stable beliefs become more easily updated (Carhart-Harris and Friston, 2019). Furthermore, bottom-up information that is normally inhibited by compressive beliefs becomes liberated and is allowed to “travel up the (brain-body) hierarchy with greater latitude and compass” (Carhart-Harris and Friston, 2019). A central characteristic of this state is increased context sensitivity, i.e., a heightened susceptibility toward ongoing processes in the internal and external context. The hypnosis session then becomes a catalyst creating a unique opportunity to modulate behavioral activation in order to promote a functional homeostasis (Greenway et al., 2020). We propose that all the mentioned findings on mechanisms involved in hypnotic analgesia are in fact not contradictory, but instead pointing toward a common ground – the role of stress and expectancies.

## Conclusion

Acute pain after breast cancer surgery is expected and adaptive, while the development from acute to CPSP represents a highly prevalent and significant clinical problem. Overall, CPSP is a multifaceted syndrome involving physiological, cognitive, and emotional factors (in addition to important socioeconomic aspects, which have not been discussed here). Expectancy effects are well-established in pain research, showing how expectancies strongly modulate acute and experimental pain. By applying CATS and principles from predictive coding framework, this review has argued how expectancies might contribute to chronic pain, in the specific case of CPSP following breast cancer surgery – mediated by sustained activation, inflammatory-induced sickness behavior, sensitization, and the neurotoxic effects of stress hormones. Clinical hypnosis is suggested as an effective intervention strategy targeting response outcome expectancies, with the potential of preventing CPSP in women with breast cancer.

## Author Contributions

HJ conceived the idea to the manuscript. AM and SR provided critical intellectual input to the disposition and conceptual framework. AM performed the literature review and wrote the first draft of the manuscript. All authors contributed to the conceptualization, writing, and approval of the final manuscript.

### Conflict of Interest

The authors declare that the research was conducted in the absence of any commercial or financial relationships that could be construed as a potential conflict of interest.
